# Development and validation of a nomogram-based prediction model for hospital-acquired carbapenem-resistant Acinetobacter baumannii in critically ill patients: a multicenter retrospective cohort study

**DOI:** 10.3389/fcimb.2025.1679272

**Published:** 2025-11-26

**Authors:** Wenjing Jiang, Anna Dai, Li Cao, Min Feng, Ping Zhou, Jun Cheng, Yan Tang, Xianglin Luo, Juan Tang

**Affiliations:** 1 Department of Hospital Infection Management, Zigong First People’s Hospital, Zigong, China; 2 Department of Nursing, Sichuan Vocational College of Health and Rehabilitation, Zigong, China; 3 Department of Hospital Infection Management, Fushun People’s Hospital, Zigong, China; 4 Department of Information Technology, Zigong First People’s Hospital, Zigong, China; 5 Department of Infectious Diseases, Zigong First People’s Hospital, Zigong, China

**Keywords:** carbapenem-resistant Acinetobacter baumannii, hospital-acquired infection, intensive care unit, nomogram, risk prediction model, multidrug-resistant organisms

## Abstract

**Background:**

Carbapenem-resistant Acinetobacter baumannii (CRAB) remains a major challenge in intensive care units (ICUs), posing substantial risks for colonization, infection, and transmission. Timely identification of patients at risk for hospital-acquired CRAB is essential to guide infection prevention and control efforts. This study aimed to develop and internally validate a nomogram for individualized risk prediction of hospital-acquired CRAB colonization or infection among critically ill patients.

**Methods:**

A retrospective multicenter cohort study was performed including 7,060 ICU patients admitted to two tertiary hospitals in China between January 1, 2019 and December 31, 2024. Candidate predictors were identified through univariate logistic regression and further refined using multivariate logistic regression with backward stepwise selection. A nomogram was subsequently constructed based on the final regression model to predict individualized risk of hospital-acquired CRAB. Model performance was evaluated in separate training and validation cohorts using area under the receiver operating characteristic curve (AUC), calibration plots, and decision curve analysis (DCA).

**Results:**

Hospital-acquired CRAB colonization or infection was observed in 224 patients (3.17%). Six independent risk factors were retained in the final model: carbapenem exposure, presence of other multidrug-resistant organisms (MDROs), mechanical ventilation, number of ICU admissions, ICU length of stay, and hospital length of stay. The nomogram exhibited strong discriminative capacity (AUC = 0.824 in the training cohort; 0.789 in the validation cohort) and demonstrated good calibration across both cohorts. DCA indicated a consistent net clinical benefit across a wide range of threshold probabilities. A risk cutoff of 0.022 (derived from the Youden index) was selected to prioritize sensitivity for infection prevention in this low-incidence setting.

**Conclusion:**

This internally validated nomogram provides an accessible tool for early identification of ICU patients at elevated risk of hospital-acquired CRAB colonization or infection. Its integration into clinical practice may facilitate risk-based prevention strategies. Future research should focus on prospective external validation and integration of environmental surveillance and microbiological genomic data to enhance the model’s predictive accuracy and generalizability.

## 1 Introduction

Carbapenem-resistant Acinetobacter baumannii (CRAB) has become one of the most formidable multidrug-resistant pathogens globally, especially in intensive care units (ICUs), where patients are highly vulnerable due to severe underlying conditions, prolonged hospital stays, frequent invasive procedures, and extensive use of broad-spectrum antibiotics (WHO, 2024; Xiong et al., 2023; Kong et al., 2025; Shi et al., 2025; Boutzoukas and Doi, 2025). CRAB infections are associated with markedly increased morbidity, mortality, and healthcare costs, primarily owing to limited therapeutic options and widespread antimicrobial resistance (Zhang et al., 2022; Li et al., 2024b). The World Health Organization (WHO) and Centers for Disease Control and Prevention (CDC) classify CRAB as a critical priority pathogen requiring urgent and coordinated action (WHO, 2024; CDC, 2025a).

In China and many middle-income countries, the prevalence of CRAB colonization and infection in ICUs has escalated dramatically over recent years, with detection rates reported as high as around 22% in some tertiary hospitals (Zhao et al., 2024; Kanani and Mullan, 2024). CRAB’s exceptional ability to persist in hospital environments and rapidly acquire resistance determinants facilitates its dissemination in high-risk settings, complicating infection control and adversely impacting patient outcomes (Xiong et al., 2023; Lu et al., 2024). Notably, CRAB colonization, often asymptomatic, is increasingly recognized as a key precursor to clinical infection and a major reservoir for nosocomial transmission (Cogliati Dezza et al., 2023; Peghin et al., 2024). Early identification of patients at risk for CRAB carriage—including both colonization and infection—is therefore crucial for timely preventive interventions.

A review of the literature shows that most prior studies have focused primarily on CRAB infection, while comparatively few have examined the high risk of progression to infection among colonized patients and their role as potential reservoirs for pathogen transmission (Cogliati Dezza et al., 2023; Peghin et al., 2024). Moreover, the majority of existing studies have not clearly distinguished between community-acquired and hospital-acquired CRAB cases, limiting their applicability for hospital infection prevention (Li et al., 2024c; Chen et al., 2024). This distinction is essential because hospital-acquired CRAB reflects exposures occurring during hospitalization—such as invasive devices, antimicrobial usage, and environmental contamination—which are amenable to targeted infection control interventions (Meschiari et al., 2021; Zhang et al., 2025b). In contrast, community-acquired CRAB often arises from prior healthcare encounters or regional antimicrobial resistance patterns, which are less immediately modifiable by hospital-based measures (Chen et al., 2024; Li et al., 2024c). Thus, focusing on hospital-acquired CRAB enables more actionable risk stratification and optimization of infection prevention resources.

Despite growing awareness of the need for accurate risk prediction, existing models for CRAB acquisition remain limited by small sample sizes, single-center designs, lack of validation, and absence of intuitive visual tools such as nomograms (Wang et al., 2020; Li et al., 2024b). Furthermore, few incorporate both colonization and infection or rigorously define hospital-acquired status, restricting clinical utility (Wang et al., 2020; Zheng et al., 2021). To address these gaps, we performed a large-scale, multicenter retrospective cohort study using electronic health record data from two tertiary hospitals in China. Our goal was to develop and internally validate a nomogram-based risk prediction model for hospital-acquired CRAB—including both colonization and infection—among critically ill patients.

## 2 Methods

### 2.1 Study design and setting

This multicenter retrospective cohort study was conducted in the central ICUs of two tertiary general hospitals [The Zigong First People’s Hospital (Center 1) and The Fushun People’s Hospital (Center 2)] in Sichuan Province, China, between January 1, 2019, and December 31, 2024.The study protocols were approved by the institutional review boards of both hospitals (Approval Nos. 2024111 and 2025KY004HY). Given the retrospective design and the use of de-identified electronic health records, the requirement for informed consent was waived. All study procedures complied with the Declaration of Helsinki and applicable national regulations. This study was reported in accordance with the Strengthening the Reporting of Observational Studies in Epidemiology (STROBE) statement for cohort studies, supplemented by relevant elements from the Standards for the Reporting of Diagnostic Accuracy Studies (STARD) where applicable (von Elm et al., 2007; Bossuyt et al., 2015).

### 2.2 Study participants

**Figure 1:**
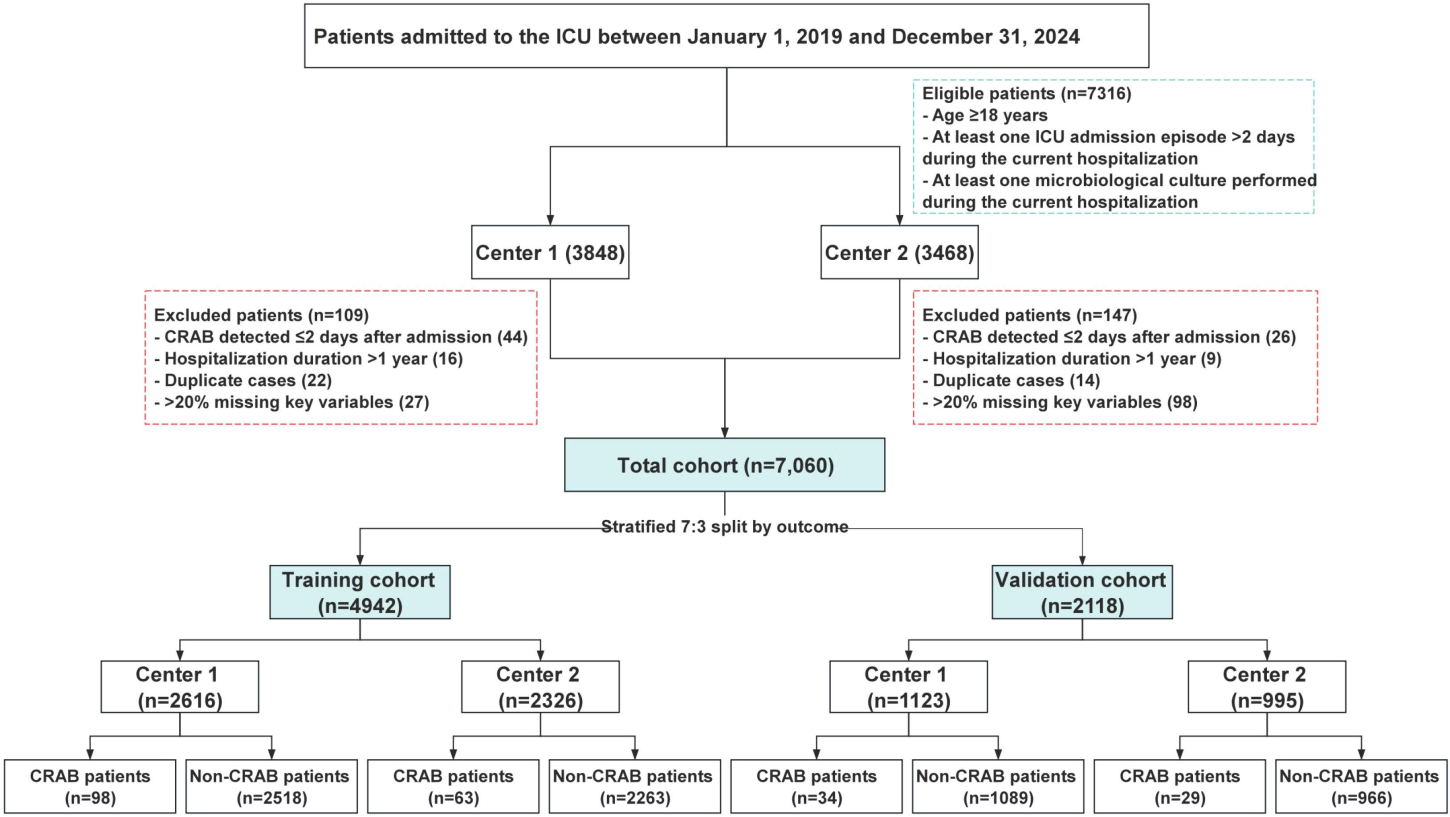
Flow chart of patient inclusion. ICU, Intensive care units; CRAB, Carbapenem-resistant Acinetobacter baumannii.

All adult patients (aged ≥18 years) admitted to the ICU during the study period were screened. Inclusion criteria were: (1) ICU length of stay > 2 calendar days and (2) at least one microbiological culture performed during hospitalization. The >2-day ICU stay criterion was applied to ensure a sufficient observation window for the assessment of exposure variables and the occurrence of hospital-acquired events, aligning with standard surveillance definitions for healthcare-associated infections (CDC, 2025b). The requirement for at least one microbiological culture was essential to ascertain the CRAB status (the outcome) for all included patients, which is a fundamental prerequisite for constructing the prediction model. Exclusion criteria included: (1) CRAB detection within the first two calendar days of admission (suggesting community-acquired or pre-existing infection); (2) hospital stay >365 days; (3) readmission within 2 days after discharge; or (4) missing >20% of key variables. After applying these criteria, a total of 7,060 eligible ICU patients were included. The cohort was randomly divided into a training set (n = 4,942) and a validation set (n = 2,118) using a 7:3 ratio, stratified by the outcome. A detailed flowchart of participant inclusion and exclusion is presented in **Figure 1**.

### 2.3 Data collection for predictors and outcome

Clinical and microbiological data were retrospectively extracted from the electronic health record systems of two tertiary hospitals using a standardized electronic case report form. Data were collected by a trained team comprising infection control practitioners and data engineers. All records underwent dual independent review and cross-validation to ensure data quality. Patient identifiers were anonymized prior to analysis to maintain confidentiality.

Candidate predictors were selected based on literature review (Meschiari et al., 2021; Cogliati Dezza et al., 2023; Peghin et al., 2024), expert consultation, and clinical judgment. The selected variables were categorized as follows: (1) demographic characteristics, including age and sex; (2) clinical severity and comorbidities, severity of illness at ICU admission was assessed using the Acute Physiology and Chronic Health Evaluation II (APACHE II) score; (3) comorbidities included hypertension, diabetes mellitus, coronary artery disease, chronic pulmonary disease, chronic kidney disease, cerebrovascular disease, and malignancy; (4) antibiotic exposure and microbiological status, including use of antibiotics (any class), use of carbapenems, and the presence of other multidrug-resistant organisms (MDROs) detected during hospitalization but before CRAB identification; (5) invasive procedures and surgical history, including central venous catheterization, mechanical ventilation, urinary catheterization, and surgical procedures performed during the hospital stay and initiated prior to CRAB detection; (6) healthcare utilization and hospitalization metrics, including history of readmission within 30 days, number of intra-hospital transfers, number of ICU admissions, length of ICU stay, and hospital length of stay.

The primary outcome was the occurrence of hospital-acquired CRAB, defined as colonization or infection with Acinetobacter baumannii resistant to carbapenems, isolated from a clinical specimen collected after the second calendar day of hospital admission (i.e., on hospital day 3 or later). CRAB colonization was defined by a positive culture in the absence of clinical signs or symptoms attributable to the organism. CRAB infection was defined by a positive culture accompanied by compatible clinical signs and symptoms, in accordance with standard CDC definitions (CDC, 2025b). Microbiological identification and antimicrobial susceptibility testing (AST) were performed using the VITEK 2 Compact system (bioMérieux, France). The requisite materials were obtained from commercial suppliers as follows: culture media (Autobio Diagnostics Co., Ltd., China), aerobic/anaerobic blood culture bottles (BD, USA), and AST-specific cards including the AST-N panels (bioMérieux, France). Antimicrobial susceptibility interpretation was performed according to the Clinical and Laboratory Standards Institute (CLSI) guidelines (M100 Edition 34, 2024).

### 2.4 Sample size considerations

Among the 7,060 included patients, 224 (3.17%) developed hospital-acquired CRAB. The sample size was deemed sufficient based on guidance for clinical prediction models. According to Riley et al (Riley et al., 2020), a minimum of 10–20 outcome events per predictor parameter (EPV) is recommended for reliable logistic regression modeling. Given that fewer than 10 variables were included in the final model and over 200 events were available, the EPV exceeded 20, satisfying recommended thresholds. Furthermore, the sample size allowed for an adequately powered training and validation cohort split.

### 2.5 Statistical methods

All statistical analyses and data visualizations were performed using R (version 4.4.2) and DCPM software (version 6.12.1, Jingding Medical Technology Co., Ltd.), which is built on the R environment. Missing data in the APACHE II score (6.5% missing) were handled using a robust approach: Multiple Imputation by Chained Equations (MICE) with m = 5 imputations, a method advised when data missingness exceeds 5%. Continuous variables were summarized as mean ± standard deviation or median with interquartile range, while categorical variables were expressed as frequencies and percentages. Group comparisons were conducted using the t-test, Mann–Whitney Utest, or Chi-square test, depending on data type and distribution. A two-sided Pvalue < 0.05 was considered statistically significant.

To develop the prediction model, univariate logistic regression was first conducted in the training cohort to identify candidate variables associated with hospital-acquired CRAB. A backward stepwise selection method was employed, guided by clinical relevance, to retain statistically significant independent predictors (P< 0.05) for inclusion in the final model. A nomogram was developed using the regression coefficients of the selected variables to enable individualized risk prediction. Internal validation was performed using bootstrap resampling with 1000 replicates to correct for overfitting and obtain optimism-adjusted performance estimates. Model discrimination was evaluated by calculating the area under the receiver operating characteristic (ROC) curve (AUC), and calibration was assessed using calibration plots. Decision curve analysis (DCA) was used to estimate the net clinical benefit across a range of threshold probabilities, thereby supporting the practical utility of the model in ICU settings. Model transportability was assessed through center-wise performance analysis and leave-one-center-out (LOCO) cross-validation, as recommended for multicenter prediction models.

## 3 Results

### 3.1 Baseline characteristics

The baseline characteristics of patients in the training and validation cohorts are presented in **Table 1**. The two cohorts were well-balanced across all assessed variables, including age, sex, APACHE II score, comorbidities, antibiotic exposure, invasive procedures, and hospital course indicators. No statistically significant differences were observed, confirming the comparability of the cohorts for model development and validation.

**Table 1:** Patients’ characteristics.

Variable	Training cohort (n=4942) M (P25, P75)/N (%)	Validation cohort (n=2118) M (P25, P75)/N (%)	*P*
Age (years)	70.00 [59.00;79.00]	71.00 [60.00;79.00]	0.102
Sex			1.000
Female	1945 (39.36%)	833 (39.33%)	
Male	2997 (60.64%)	1285 (60.67%)	
APACHE II score	26.00 [19.00;33.00]	26.00 [19.00;32.00]	0.135
Hypertension			0.746
No	3374 (68.27%)	1437 (67.85%)	
Yes	1568 (31.73%)	681 (32.15%)	
Diabetes mellitus			0.593
No	3855 (78.00%)	1665 (78.61%)	
Yes	1087 (22.00%)	453 (21.39%)	
Coronary artery disease			0.449
No	3864 (78.19%)	1638 (77.34%)	
Yes	1078 (21.81%)	480 (22.66%)	
Chronic pulmonary disease			0.550
No	3602 (72.89%)	1559 (73.61%)	
Yes	1340 (27.11%)	559 (26.39%)	
Chronic kidney disease			0.495
No	4105 (83.06%)	1774 (83.76%)	
Yes	837 (16.94%)	344 (16.24%)	
Cerebrovascular disease			0.534
No	3723 (75.33%)	1611 (76.06%)	
Yes	1219 (24.67%)	507 (23.94%)	
Malignancy			0.292
No	4502 (91.10%)	1912 (90.27%)	
Yes	440 (8.90%)	206 (9.73%)	
Use of antibiotics			0.215
No	391 (7.91%)	187 (8.83%)	
Yes	4551 (92.09%)	1931 (91.17%)	
Use of carbapenems			0.699
No	3849 (77.88%)	1640 (77.43%)	
Yes	1093 (22.12%)	478 (22.57%)	
Presence of other MDROs			0.863
No	4851 (98.16%)	2077 (98.06%)	
Yes	91 (1.84%)	41 (1.94%)	
Central venous catheterization			0.903
No	3358 (67.95%)	1443 (68.13%)	
Yes	1584 (32.05%)	675 (31.87%)	
Mechanical ventilation			0.450
No	2824 (57.14%)	1189 (56.14%)	
Yes	2118 (42.86%)	929 (43.86%)	
Urinary catheterization			0.915
No	106 (2.14%)	47 (2.22%)	
Yes	4836 (97.86%)	2071 (97.78%)	
Surgery			0.501
No	3624 (73.33%)	1536 (72.52%)	
Yes	1318 (26.67%)	582 (27.48%)	
30 day readmission history			0.228
No	4359 (88.20%)	1890 (89.24%)	
Yes	583 (11.80%)	228 (10.76%)	
Number of intra hospital transfers			0.813
0	1444 (29.22%)	631 (29.79%)	
1	1760 (35.61%)	751 (35.46%)	
2	1256 (25.41%)	544 (25.68%)	
3	482 (9.75%)	192 (9.07%)	
Number of ICU admissions			1.000
1	4771 (96.54%)	2045 (96.55%)	
≥2	171 (3.46%)	73 (3.45%)	
ICU length of stay (days)	6.00 [4.00;9.00]	6.00 [4.00;9.00]	0.835
Hospital length of stay (days)	14.00 [9.00;23.00]	14.00 [9.00;23.00]	0.763

### 3.2 Distribution of specimen sources

The vast majority of initial CRAB-positive specimens (78.6%, 176/224) were from the respiratory tract (sputum or bronchoalveolar lavage fluid), while 15.6% (35/224) were from blood, and the remaining 5.8% (13/224) were from other sites. This distribution is consistent with the established epidemiology of CRAB in ICU settings, highlighting the respiratory tract as the primary site of detection.

### 3.3 Predictors of outcome

Univariate logistic regression analysis was performed in the training cohort to identify potential predictors of hospital-acquired CRAB colonization or infection. Variables with a P value < 0.05 were considered candidates for multivariate analysis. These included the APACHE II score, use of any antibiotics, carbapenem exposure, presence of other MDROs, central venous catheterization, mechanical ventilation, number of ICU admissions, ICU length of stay, and hospital length of stay (**Table 2**).

**Table 2:** Univariate and multivariate analyses for factors associated with hospital-acquired CRAB.

Variable	Univariate analysis		Multivariate analysis		
	OR (95% CI)	*P*	Coefficient (β)	OR (95% CI)	*P*
Intercept	–	*-*	-5.387	–	*-*
Age	1.007(0.996-1.019)	0.197			
Sex	1.374(0.988-1.935)	0.063			
APACHE II score	1.034(1.018-1.050)	<0.001			
Hypertension	1.186(0.849-1.641)	0.309			
Diabetes mellitus	1.139(0.780-1.628)	0.488			
Coronary artery disease	1.151(0.788-1.646)	0.452			
Chronic pulmonary disease	1.112(0.780-1.561)	0.547			
Chronic kidney disease	1.378(0.928-1.996)	0.100			
Cerebrovascular disease	1.154(0.803-1.629)	0.426			
Malignancy	1.300(0.763-2.087)	0.304			
Central venous catheterization	2.386(1.741-3.274)	<0.001			
Use of antibiotics	14.210(3.181-250.200)	<0.001			
Use of carbapenems	5.760(4.180-7.989)	<0.001	1.360	3.897 (2.765–5.493)	<0.001
Presence of other MDROs	10.800(6.373-17.70)	<0.001	1.279	3.592 (1.999–6.455)	<0.001
Mechanical ventilation	3.361(2.398-4.785)	<0.001	0.710	2.033 (1.407–2.939)	<0.001
Urinary catheterization	1.768(0.554-10.77)	0.428			
Surgery	0.969(0.671-1.374)	0.865			
30 day readmission history	1.191(0.734-1.843)	0.456			
Number of intra hospital transfers	1.108(0.941-1.302)	0.217			
Number of ICU admissions	4.349(2.580-6.986)	<0.001	0.729	2.074 (1.200–3.585)	0.009
ICU length of stay	1.017(1.013-1.022)	<0.001	0.008	1.008 (1.002–1.013)	0.006
Hospital length of stay	1.009(1.007-1.011)	<0.001	0.004	1.004 (1.000–1.007)	0.043

Subsequently, multivariate logistic regression identified six independent risk factors: carbapenem exposure, presence of other MDROs, mechanical ventilation, number of ICU admissions, ICU length of stay, and hospital length of stay, all of which remained statistically significant (P < 0.05) (**Table 2**). These factors were incorporated into the final prediction model.

### 3.4 Nomogram construction

**Figure 2:**
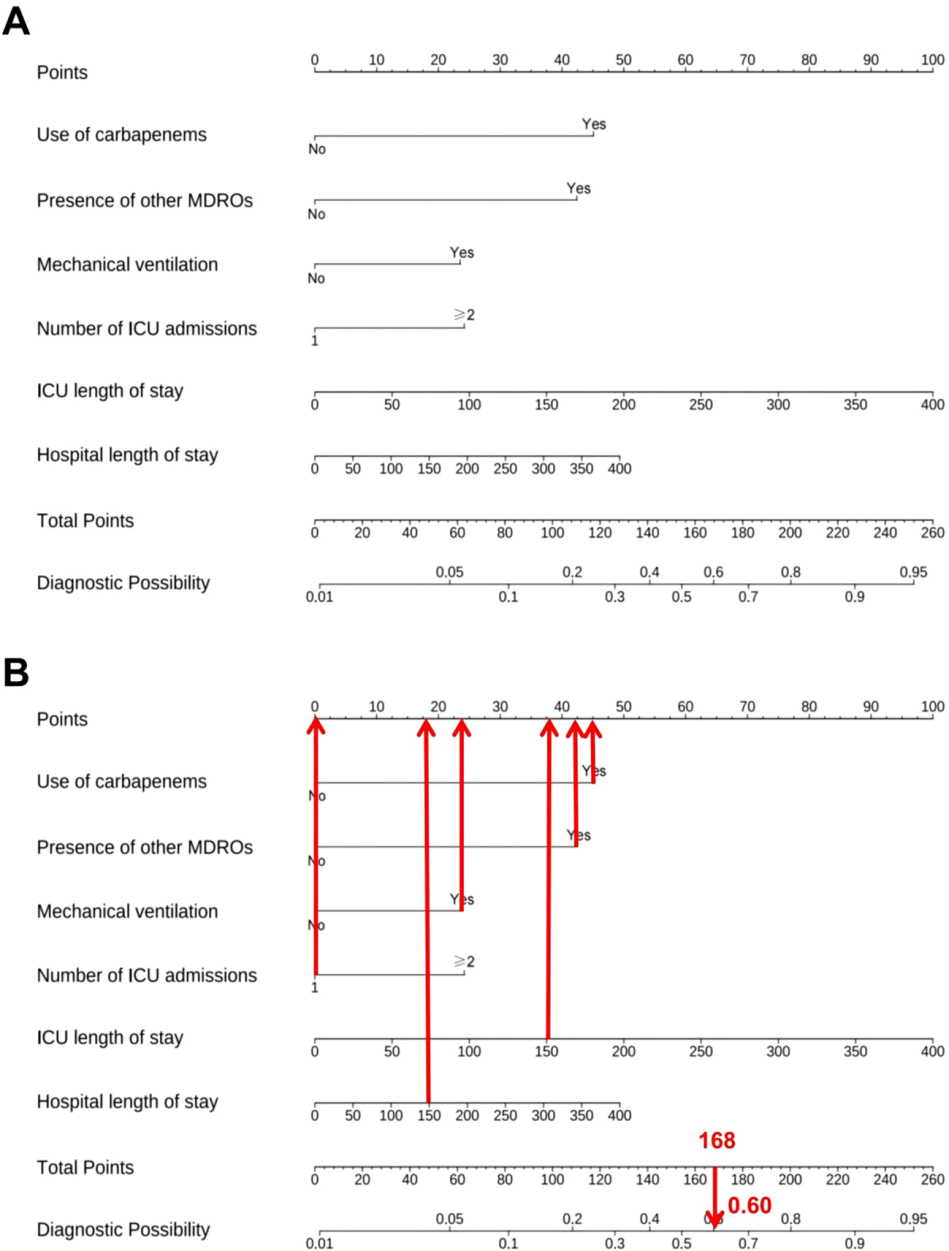
Risk prediction model for hospital-acquired CRAB. **(A)** Nomogram. **(B)** Clinical application example.

A predictive nomogram was developed based on the six independent predictors identified through multivariate logistic regression analysis (**Figures 2A, B**). Each variable was assigned a weighted point score derived from its corresponding regression coefficient, and the total cumulative score was translated into an estimated probability of hospital-acquired CRAB. To illustrate the clinical application of the nomogram, a representative case was evaluated: an ICU patient with carbapenem exposure, presence of other MDROs, mechanical ventilation, one ICU admission, an ICU length of stay of 150 days, and a hospital length of stay of 150 days. This patient profile yielded a total score of 168, corresponding to an estimated hospital-acquired CRAB risk of 60%. The nomogram thus serves as a visual tool for the early estimation of individualized risk based on specific patient factors. The complete and validated R implementation is provided in **Supplementary File S1**.

### 3.5 Nomogram validation

**Figure 3:**
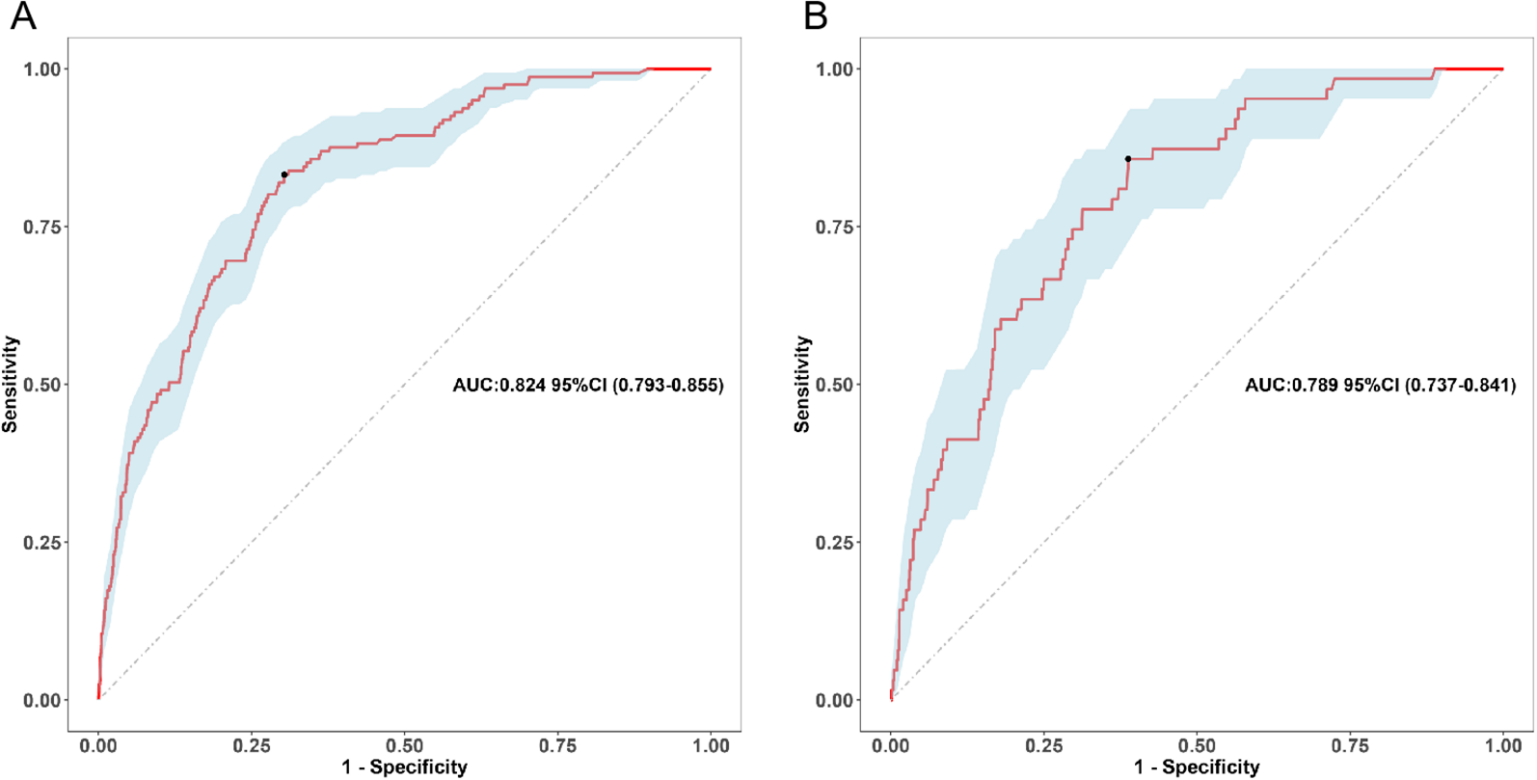
Receiver operating characteristic (ROC) curve of the nomogram. **(A)** Training cohort, **(B)** Validation cohort.

The nomogram demonstrated strong discriminative ability in the derivation cohort. The area under the receiver operating characteristic curve (AUC) was 0.824 (95% CI: 0.793–0.855) in the training set and 0.789 (95% CI: 0.737–0.841) in the validation set (**Figures 3A, B**), indicating robust predictive accuracy. Bootstrap internal validation (1000 replicates) was conducted to assess model stability and adjust for potential overfitting. The optimism-corrected performance metrics aligned closely with the apparent performance, indicating robust internal validity (**Supplementary Figures 1A, B**).

**Figure 4:**
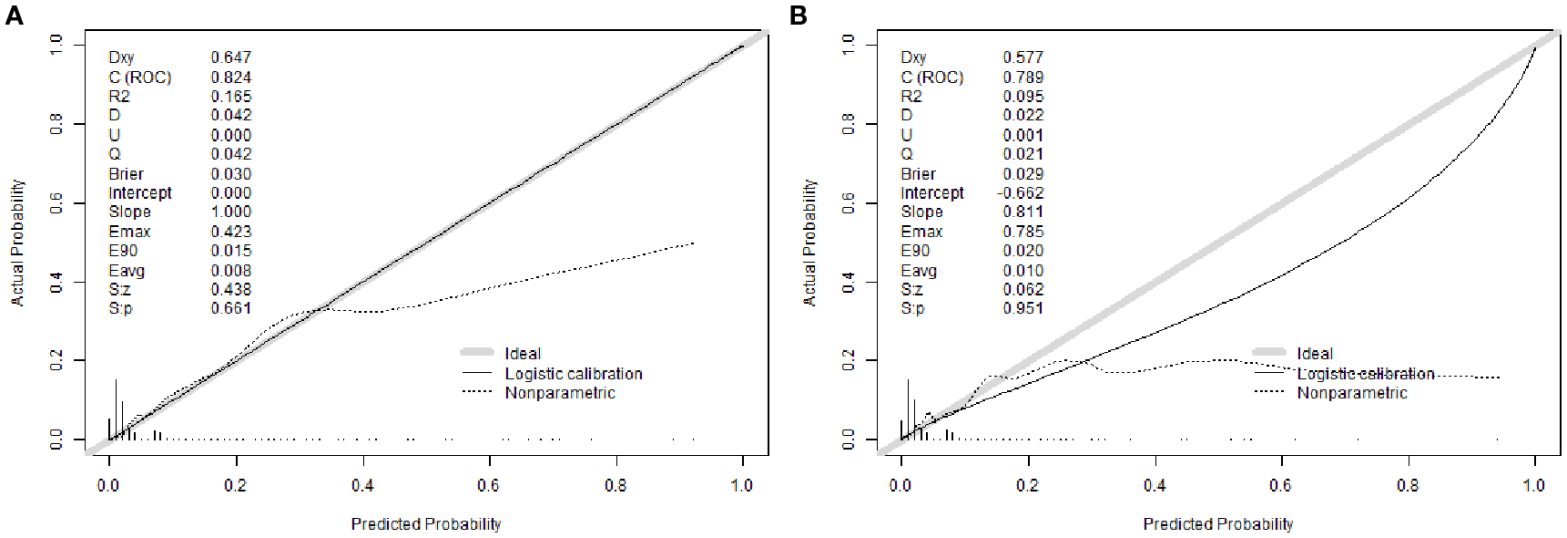
Calibration curves of the nomogram. **(A)** Training cohort, **(B)** Validation cohort.

Calibration analysis revealed excellent agreement between predicted probabilities and observed outcomes. The calibration intercept was 0.000 with a slope of 1.000 in the training cohort, indicating near-perfect calibration. In the validation cohort, the intercept was -0.662 with a slope of 0.811, suggesting acceptable calibration with minor systematic underestimation of risk. The Brier scores were 0.030 (training) and 0.029 (validation), confirming high overall prediction accuracy (**Figures 4A, B**).

To evaluate model transportability and robustness across clinical settings, we performed two complementary validation strategies: center-wise performance analysis and leave-one-center-out (LOCO) cross-validation. The discriminative performance varied between centers, with AUC values of 0.502 (95% CI: 0.467–0.537) for Center 1 and 0.568 (95% CI: 0.529–0.607) for Center 2 (**Table 3**). The LOCO validation yielded an average AUC of 0.535 (**Table 4**), representing a -9.5% relative change from the overall performance. This observed heterogeneity in model performance across centers and the performance decrement in LOCO analysis highlight the challenge of maintaining predictive accuracy when transitioning between healthcare settings with distinct patient populations and clinical protocols. These variations likely reflect differences in local case-mix, clinical practices, and CRAB endemicity levels between the participating institutions.

**Table 3:** Center-wise performance of the CRAB prediction model.

Center	Samples, n	Events, n (%)	AUC (95% CI)	Calibration Slope (95% CI)
1	3739	132(3.5%)	0.502(0.467-0.537)	-1.29(-1.68 to -0.90)
2	3321	92(2.8%)	0.568(0.529-0.607)	-19.57(-25.13 to -14.01)
Overall	7060	224(3.2%)​	0.524(0.503-0.545)	-10.43(-13.21 to -7.65)​

Performance metrics were calculated for each participating center independently. The overall performance was obtained by pooling all data. Calibration slope values significantly different from 1.0 (perfect calibration) indicate miscalibration.

**Table 4:** Leave-One-Center-Out (LOCO) cross-validation results.

Excluded center	AUC (95% CI)	Calibration intercept	Calibration slope	Performance change
1	0.502 (0.467 - 0.537)	-3.26	-1.29	-4.20%
2	0.568 (0.529 - 0.607)	-3.16	-19.57	8.40%
Average	0.535 (0.515 - 0.555)	-3.21	-10.43	+2.1%

Performance change calculated as: (LOCO AUC - Overall AUC)/Overall AUC × 100%. Positive values indicate better performance on unseen centers, negative values indicate worse performance. LOCO validation assesses model transportability by iteratively excluding each center and evaluating performance on the held-out center.

**Figure 5:**
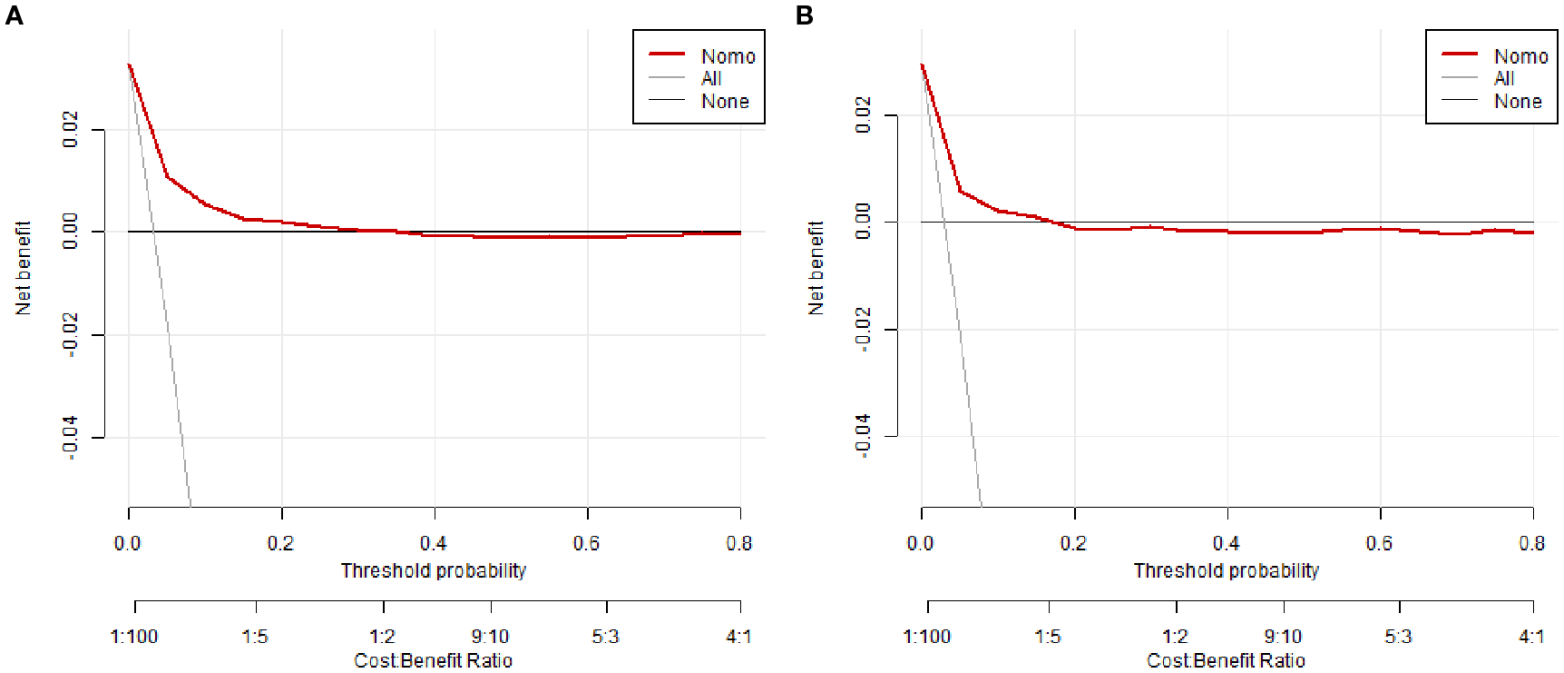
Decision curve analysis (DCA) of the nomogram. **(A)** Training cohort, **(B)** Validation cohort.

To evaluate clinical utility, DCA was performed (**Figures 5A, B**; **Supplementary Figures 2A, B**). The nomogram consistently showed superior net benefit across a wide range of threshold probabilities (0%–80%) compared to default strategies of treating all or no patients. In the clinically relevant range (20%–30%), the model significantly reduced overtreatment while preserving high sensitivity in risk classification.

The optimal probability threshold for identifying high-risk individuals was determined to be 0.022, based on the maximum Youden index on the ROC curve (**Figure 3A**). Individuals with predicted probabilities exceeding this threshold were classified as high-risk for hospital-acquired CRAB. The diagnostic metrics, including accuracy, sensitivity, specificity, positive predictive value, and negative predictive value, are detailed for both the training and validation cohorts in **Table 5**.

**Table 5:** Model performance.

Cohort	Accuracy	Sensitivity	Specificity	Positive predictive value	Negative predictive value
Training cohort	0.692 (0.692-0.692)	0.839 (0.782-0.895)	0.687 (0.674-0.700)	0.083 (0.069-0.096)	0.992 (0.989-0.995)
Validation cohort	0.674 (0.674-0.674)	0.778 (0.675-0.880)	0.671 (0.651-0.691)	0.068 (0.049-0.086)	0.990 (0.985-0.995)

Data presented as point estimate (lower limit-upper limit) of 95% confidence interval. Cutoff value = 0.022.

## 4 Discussion

In this multicenter retrospective cohort study involving critically ill patients from two tertiary hospitals in China, we developed and internally validated a nomogram-based prediction model for hospital-acquired CRAB colonization and infection. Our model incorporates six independent clinical risk factors—including carbapenem exposure, presence of other MDROs, mechanical ventilation, number of ICU admissions, and length of ICU and hospital stays—and demonstrates robust discrimination (AUC 0.824 in the training cohort and 0.789 in the validation cohort) alongside excellent calibration. This pragmatic and visually intuitive tool holds promise for early identification and precise risk stratification of patients susceptible to hospital-acquired CRAB, thus supporting tailored infection prevention and antimicrobial stewardship strategies within ICU settings.

Our findings reinforce and expand upon existing evidence that antimicrobial exposure and invasive procedures constitute principal drivers of CRAB acquisition (Boutzoukas and Doi, 2025; Zhang et al., 2025b). Carbapenem use exerts potent selective pressure that disrupts the host microbiota’s ecological balance, fostering the expansion and colonization of resistant strains (Gedefie et al., 2023; Khosravy et al., 2023; Zhang et al., 2025b). Recent studies employing metagenomic and microbiome analyses have elucidated that antibiotic-induced dysbiosis diminishes colonization resistance and facilitates horizontal gene transfer among microbial communities, thereby accelerating resistance propagation (Gedefie et al., 2023; Khosravy et al., 2023; Zhang et al., 2025b). The frequent coexistence of other MDROs likely reflects overlapping ecological niches and intricate cross-transmission networks within healthcare environments, with polymicrobial synergism intensifying colonization pressure and facilitating pathogen dissemination (Boutzoukas and Doi, 2025; Zhang et al., 2025c). Additionally, surface contamination and biofilm formation on medical devices provide a stable reservoir that promotes persistent colonization and complicates eradication efforts (Khosravy et al., 2023; Xiong et al., 2023; Kong et al., 2025).Mechanical ventilation, a common invasive intervention in ICUs, compromises respiratory epithelial integrity and triggers localized inflammation, which collectively enhance bacterial adherence and biofilm development on respiratory devices (Zhang et al., 2021; Khosravy et al., 2023; Novović et al., 2023). This biofilm not only protects CRAB from host immune defenses but also increases antimicrobial tolerance, resulting in persistent colonization and heightened infection risk. Such insights underscore the critical need to optimize ventilator care and explore anti-biofilm strategies to mitigate CRAB transmission.

Beyond these established risk factors, our evaluation revealed substantial heterogeneity in model performance across participating centers, with discriminative ability (AUC) ranging from 0.502 to 0.568. This observed variability underscores several important methodological and clinical considerations. First, the performance differences likely reflect fundamental variations in local case-mix, including differences in patient acuity, comorbidity profiles, and CRAB endemicity levels between institutions. Second, variations in clinical practices—including antibiotic stewardship protocols, infection control measures, and microbiological testing frequency—may significantly influence both CRAB risk and model performance. Third, the LOCO validation demonstrated a -9.5% decrement in AUC when applied to unseen centers, highlighting the challenge of maintaining predictive accuracy across diverse healthcare environments. This heterogeneity provides a critical context for interpreting the following dose-dependent association between healthcare exposure and risk.

Notwithstanding the observed inter-center performance variability, a consistent and significant dose-dependent association emerged between cumulative healthcare exposure—reflected by multiple ICU admissions and prolonged stays—and CRAB acquisition risk. This observation aligns with prior evidence that extended and repeated hospitalizations exacerbate colonization likelihood, potentially through immune impairment, microbiota alterations, and increased exposure to contaminated environments (Qiao et al., 2020; Wu et al., 2023). These findings highlight the limitations of static admission-based risk assessments and advocate for dynamic, longitudinal surveillance and risk stratification models. Incorporating time-series clinical data and leveraging machine learning algorithms could enhance predictive accuracy and enable proactive infection control measures.

Uniquely, our study integrates both CRAB colonization and infection as outcomes, applying a stringent definition of hospital-acquired cases based on specimen collection after hospital day two. This approach addresses gaps in previous research that often conflated community- and hospital-acquired cases or limited focus to clinical infection, thereby refining risk factor elucidation and enhancing the precision of targeted interventions (Cogliati Dezza et al., 2023; Peghin et al., 2024). Recognizing colonization as a pivotal precursor state is increasingly supported by evidence demonstrating its role as a reservoir facilitating horizontal transmission and progression to invasive disease (Cogliati Dezza et al., 2023; Peghin et al., 2024).

The constructed nomogram utilizes routinely collected clinical variables to provide a user-friendly bedside risk assessment tool. Despite observed performance variations across centers, the model maintained clinical utility in decision curve analysis, providing a foundation for localized implementation following center-specific calibration and validation. DCA demonstrates that our model provides superior net benefit compared to “treat-all” or “treat-none” strategies across a wide range of threshold probabilities, reinforcing its clinical utility. It is important to note that the optimal cutoff of 0.022, while statistically robust, yields a relatively low positive predictive value (PPV) given the low incidence (~3%) of the outcome. Therefore, the ‘high-risk’ designation is best interpreted as a trigger for enhanced infection prevention protocols (e.g., contact precautions) rather than as a direct guide for therapeutic decisions, a distinction well-supported by the net benefit shown in the DCA. Compared with previously published models—often limited by small sample sizes, single-center biases, or lack of calibration assessment—our model benefits from a large multicenter dataset and rigorous internal validation, enhancing generalizability within similar tertiary ICU populations (Peghin et al., 2024; Boutzoukas and Doi, 2025; Zhang et al., 2025b). Building upon these foundations, future investigations should prioritize external validation and explore the integration of this nomogram into electronic health record systems to facilitate real-time risk assessment and serve as a clinical decision support tool.

Nonetheless, several limitations warrant consideration. First, and most critically, the retrospective design limits our ability to establish a fixed, prospective prediction timepoint and introduces potential for residual confounding. Although exposure variables such as carbapenem use and mechanical ventilation were ascertained prior to CRAB detection, the precise temporal sequence and duration of these exposures remain challenging to delineate. This inherent limitation may inflate the apparent model performance and underscores the need for caution in clinical interpretation. Second, the observed heterogeneity in model performance across centers (AUC range: 0.502–0.568) underscores the challenge of developing universally applicable prediction models in heterogeneous ICU settings and highlights the need for local calibration before clinical implementation.Third, although internal validation demonstrated robustness, external validation across diverse geographic and healthcare contexts is essential to confirm generalizability (Collins et al., 2024). Fourth, the absence of critical infection control variables—such as environmental contamination metrics and healthcare worker hand hygiene adherence—may limit comprehensive capture of all acquisition drivers (Li et al., 2024a; Wei et al., 2024). Fifth, lack of molecular characterization of CRAB isolates restricts insight into clonal dissemination and resistance evolution (Xu et al., 2024). Finally, by design, our model predicts a composite outcome of CRAB acquisition without distinguishing colonization from infection. While this approach is optimal for triggering broad infection prevention measures, it does not directly guide specific therapeutic interventions, which represents a focus for future antimicrobial stewardship models.

## 5 Conclusion

Our study presents an internally validated nomogram integrating key clinical predictors for hospital-acquired CRAB colonization and infection among critically ill patients. The nomogram thereby facilitates early recognition of high-risk individuals and provides a practical tool to guide targeted infection control and antimicrobial stewardship interventions. Future research should prioritize multicenter external prospective validation and the integration of environmental surveillance and molecular genomic data to further enhance the model’s clinical utility in curbing CRAB transmission in ICU settings.

## Data Availability

The raw data supporting the conclusions of this article will be made available by the authors, without undue reservation.

## References

[B1] Bossuyt P. M. Reitsma J. B. Bruns D. E. Gatsonis C. A. Glasziou P. P. Irwig L. . (2015). STARD 2015: an updated list of essential items for reporting diagnostic accuracy studies. BMJ 351, h5527. doi: 10.1136/bmj.h5527, PMID: 26511519 PMC4623764

[B2] Boutzoukas A. Doi Y. (2025). The global epidemiology of carbapenem-resistant Acinetobacter baumannii. JAC Antimicrob. Resist. 7, dlaf134. doi: 10.1093/jacamr/dlaf134, PMID: 40735512 PMC12305305

[B3] CDC (2025a). Antimicrobial resistance facts and stats. Available online at: https://www.cdc.gov/antimicrobial-resistance/data-research/facts-stats/index.html (Accessed August 3, 2025).

[B4] CDC (2025b). Identifying HAIs for NHSN surveillance (n.d.). Available online at: https://www.cdc.gov/nhsn/pdfs/pscmanual/2psc_identifyinghais_nhsncurrent.pdf?utm_source=chatgpt.com (Accessed August 3, 2025).

[B5] Chen J. Li G. Shao Y. Cheng Z. Wan F. Wu D. . (2024). Clinical, phenotypic characterization and genomic analysis of the mucoid Acinetobacter baumannii from a teaching hospital. Microbial Pathogenesis 196, 106929. doi: 10.1016/j.micpath.2024.106929, PMID: 39270758

[B6] Cogliati Dezza F. Covino S. Petrucci F. Sacco F. Viscido A. Gavaruzzi F. . (2023). Risk factors for carbapenem-resistant Acinetobacter baumannii (CRAB) bloodstream infections and related mortality in critically ill patients with CRAB colonization. JAC Antimicrob. Resist. 5, dlad096. doi: 10.1093/jacamr/dlad096, PMID: 37577156 PMC10412853

[B7] Collins G. S. Dhiman P. Ma J. Schlussel M. M. Archer L. Calster B. V. . (2024). Evaluation of clinical prediction models (part 1): from development to external validation. BMJ 384, e074819. doi: 10.1136/bmj-2023-074819, PMID: 38191193 PMC10772854

[B8] Gedefie A. Alemayehu E. Mohammed O. Bambo G. M. Kebede S. S. Kebede B. (2023). Prevalence of biofilm producing Acinetobacter baumannii clinical isolates: A systematic review and meta-analysis. PloS One 18, e0287211. doi: 10.1371/journal.pone.0287211, PMID: 38032906 PMC10688650

[B9] Kanani D. K. Mullan D. S. (2024). Characterization of acinetobacter baumannii resistance and prevalence in icu settings: insights from A retrospective study. IOSRJDMS 23, 35–39. doi: 10.9790/0853-2309043539

[B10] Khosravy M. Hosseini F. Razavi M. R. Khavari R. A. (2023). Expression of biofilm-related genes in extensively drug-resistant acinetobacter baumannii. Jundishapur J. Microbiol. 16, e133999. doi: 10.5812/jjm-133999

[B11] Kong Y. Liu T. Zhang Y. Wang H. Lin H. (2025). Investigation of an outbreak of carbapenem resistant Acinetobacter baumannii in an intensive care unit during the COVID-19 epidemic. Antimicrobial Resistance Infection Control 14, 30. doi: 10.1186/s13756-025-01547-0, PMID: 40221801 PMC11994003

[B12] Li Y. Cao Y. Wang M. Wang L. Wu Y. Fang Y. . (2024a). Development and validation of machine learning models to predict MDRO colonization or infection on ICU admission by using electronic health record data. Antimicrob. Resist. Infect. Control 13, 74. doi: 10.1186/s13756-024-01428-y, PMID: 38971777 PMC11227715

[B13] Li Y. Gao X. Diao H. Shi T. Zhang J. Liu Y. . (2024b). Development and application of a risk nomogram for the prediction of risk of carbapenem-resistant Acinetobacter baumannii infections in neuro-intensive care unit: a mixed method study. Antimicrobial Resistance Infection Control 13, 62. doi: 10.1186/s13756-024-01420-6, PMID: 38867312 PMC11170918

[B14] Li Y. Zhang J. Gu Y. Wang L. Hu J. (2024). Nosocomial, healthcare-associated, and community-acquired acinetobacter baumannii in China: clinical characteristics, antimicrobial resistance patterns and risk factors associated with carbapenem resistance. Infect. Drug Resist. 17, 4089–4099. doi: 10.2147/IDR.S469244, PMID: 39319039 PMC11420889

[B15] Lu Y. Zhao X. Du L. Huang R. Liu Y. Ma X. . (2024). Retrospective effect evaluation of intensive disinfection on preventing cluster infection with multidrug-resistant Acinetobacter baumannii in ICU. Ster Supply 3, 134–141. doi: 10.11910/j.issn.2791-2043.2024.3.04

[B16] Meschiari M. Kaleci S. Orlando G. Selmi S. Santoro A. Bacca E. . (2021). Risk factors for nosocomial rectal colonization with carbapenem-resistant Acinetobacter baumannii in hospital: a matched case–control study. Antimicrobial Resistance Infection Control 10, 69. doi: 10.1186/s13756-021-00919-6, PMID: 33832538 PMC8028794

[B17] Novović K. Kuzmanović Nedeljković S. Poledica M. Nikolić G. Grujić B. Jovčić B. . (2023). Virulence potential of multidrug-resistant Acinetobacter baumannii isolates from COVID-19 patients on mechanical ventilation: The first report from Serbia. Front. Microbiol. 14. doi: 10.3389/fmicb.2023.1094184, PMID: 36825087 PMC9941878

[B18] Peghin M. Givone F. de Martino M. Ali R. W. Graziano E. Isola M. . (2024). Risk factors for infection after carbapenem-resistant Acinetobacter baumannii colonization. Eur. J. Clin. Microbiol. Infect. Dis. 43, 2191–2199. doi: 10.1007/s10096-024-04936-2, PMID: 39285106 PMC11534838

[B19] Qiao F. Huang W. Gao S. Cai L. Zhu S. Wei L. . (2020). Risk factor for intestinal carriage of carbapenem-resistant Acinetobacter baumannii and the impact on subsequent infection among patients in an intensive care unit: an observational study. BMJ Open 10, e035893. doi: 10.1136/bmjopen-2019-035893, PMID: 32912943 PMC7482480

[B20] Riley R. D. Ensor J. Snell K. I. E. Harrell F. E. Martin G. P. Reitsma J. B. . (2020). Calculating the sample size required for developing a clinical prediction model. BMJ 368, m441. doi: 10.1136/bmj.m441, PMID: 32188600

[B21] Shi J. Mao X. Sun F. Cheng J. Shao L. Shan X. . (2025). Epidemiological characteristics and antimicrobial resistance of extensively drug-resistant Acinetobacter baumannii in ICU wards. Microbiol. Spectr. 13, e0261924. doi: 10.1128/spectrum.02619-24, PMID: 40035537 PMC11960075

[B22] von Elm E. Altman D. G. Egger M. Pocock S. J. Gøtzsche P. C. Vandenbroucke J. P. . (2007). The Strengthening the Reporting of Observational Studies in Epidemiology (STROBE) statement: guidelines for reporting observational studies. Ann. Intern. Med. 147, 573–577. doi: 10.7326/0003-4819-147-8-200710160-00010, PMID: 17938396

[B23] Wang L. Huang X. Zhou J. Wang Y. Zhong W. Yu Q. . (2020). Predicting the occurrence of multidrug-resistant organism colonization or infection in ICU patients: development and validation of a novel multivariate prediction model. Antimicrob. Resist. Infect. Control 9, 66. doi: 10.1186/s13756-020-00726-5, PMID: 32430043 PMC7236142

[B24] Wei L. Feng Y. Lin J. Kang X. Zhuang H. Wen H. . (2024). Handwashing sinks as reservoirs of carbapenem-resistant Acinetobacter baumannii in the intensive care unit: a prospective multicenter study. Front. Public Health 12. doi: 10.3389/fpubh.2024.1468521, PMID: 39444981 PMC11496070

[B25] WHO (2024). WHO bacterial priority pathogens list 2024: Bacterial pathogens of public health importance to guide research, development and strategies to prevent and control antimicrobial resistance (n.d.). Available online at: https://www.who.int/publications/i/item/9789240093461 (Accessed August 3, 2025).

[B26] Wu Y.-L. Hu X.-Q. Wu D.-Q. Li R.-J. Wang X.-P. Zhang J. . (2023). Prevalence and risk factors for colonisation and infection with carbapenem-resistant *Enterobacterales* in intensive care units: A prospective multicentre study. Intensive Crit. Care Nurs. 79, 103491. doi: 10.1016/j.iccn.2023.103491, PMID: 37480701

[B27] Xiong L. Deng C. Yang G. Shen M. Chen B. Tian R. . (2023). Molecular epidemiology and antimicrobial resistance patterns of carbapenem-resistant Acinetobacter baumannii isolates from patients admitted at ICUs of a teaching hospital in Zunyi, China. Front. Cell. Infect. Microbiol. 13. doi: 10.3389/fcimb.2023.1280372, PMID: 38106474 PMC10722174

[B28] Xu A. Li M. Hang Y. Zeng L. Zhang X. Hu Y. . (2024). Multicenter retrospective genomic characterization of carbapenemase-producing Acinetobacter baumannii isolates from Jiangxi patients 2021–2022: identification of a novel international clone, IC11. mSphere 9, e00276–e00224. doi: 10.1128/msphere.00276-24, PMID: 38832781 PMC11332331

[B29] Zhang J. Liu W. Shi W. Cui X. Liu Y. Lu Z. . (2022). A nomogram with six variables is useful to predict the risk of acquiring carbapenem-resistant microorganism infection in ICU patients. Front. Cell. Infect. Microbiol. 12. doi: 10.3389/fcimb.2022.852761, PMID: 35402310 PMC8990894

[B30] Zhang X. Tian L. Wu J. Chen T. Shi T. Ge Y. . (2025c). Risk factors for Carbapenem-resistant Acinetobacter baumannii contamination on hospital surfaces: a multi-year environmental monitoring study in Shanghai, China. Front. Microbiol. 16. doi: 10.3389/fmicb.2025.1609148, PMID: 40611962 PMC12222070

[B31] Zhang S. Xiao J. Li Y. Li W. Li Y. Pang M. . (2025b). An integrative review on the risk factors, prevention, and control strategies for carbapenem-resistant Acinetobacter baumannii colonization in critically ill patients. Front. Microbiol. 15. doi: 10.3389/fmicb.2024.1519906, PMID: 39867493 PMC11757275

[B32] Zhang T. Xu X. Xu C.-F. Bilya S. R. Xu W. (2021). Mechanical ventilation-associated pneumonia caused by Acinetobacter baumannii in Northeast China region: analysis of genotype and drug resistance of bacteria and patients’ clinical features over 7 years. Antimicrobial Resistance Infection Control 10, 135. doi: 10.1186/s13756-021-01005-7, PMID: 34526127 PMC8444615

[B33] Zhao S. Zhang B. Liu C. Sun X. Chu Y. (2024). Acinetobacter baumannii infection in intensive care unit: analysis of distribution and drug resistance. Mol. Biol. Rep. 51, 120. doi: 10.1007/s11033-023-09144-3, PMID: 38227070

[B34] Zheng Y. Xu N. Pang J. Han H. Yang H. Qin W. . (2021). Colonization with extensively drug-resistant acinetobacter baumannii and prognosis in critically ill patients: an observational cohort study. Front. Med. (Lausanne) 8. doi: 10.3389/fmed.2021.667776, PMID: 33996866 PMC8119758

