# Supplementary material for: Development and validation of a nomogram-based prediction model for hospital-acquired carbapenem-resistant Acinetobacter baumannii in critically ill patients: a multicenter retrospective cohort study
Source: Front Cell Infect Microbiol. 2025 Nov 26;15:1679272. doi: 10.3389/fcimb.2025.1679272 (PMC12689862; doi:10.3389/fcimb.2025.1679272)
Supplement: Supplementary file 1 [file Table1.docx]

****
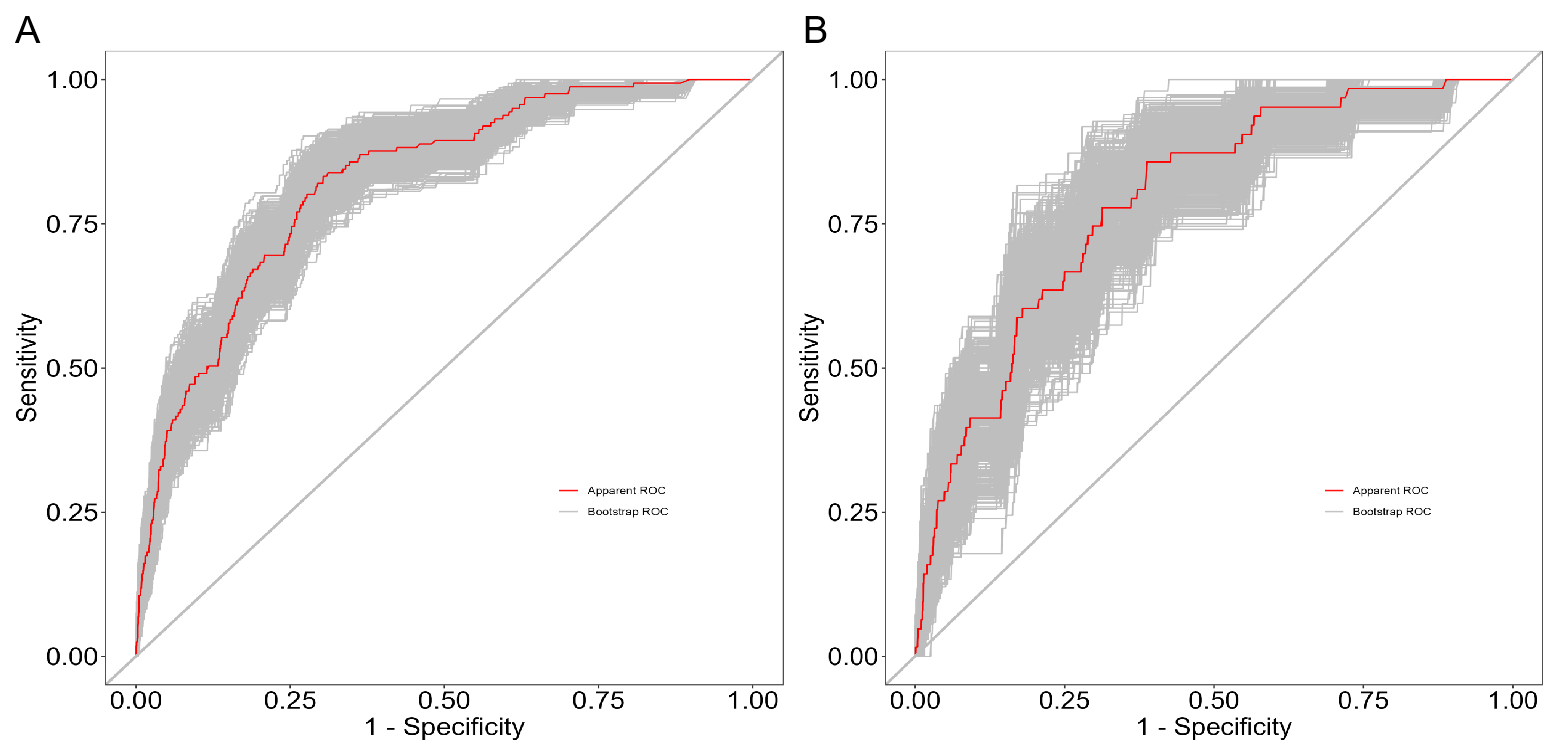
****

****Supplementary Figure 1**** Bootstrap-validated receiver operating characteristic (ROC) curves for the training and validation cohorts.​​

(A) Training cohort, (B) Validation cohort

The bootstrap internal validation demonstrated robust discriminative performance across both cohorts. In the training cohort, the optimism-corrected area under the curve (AUC) was 0.821 (95% CI: 0.792–0.851). The model maintained strong predictive accuracy in the validation cohort, with an AUC of 0.799 (95% CI: 0.743–0.855). The gray bands represent the distribution of AUC values from 1000 bootstrap replicates, which visually confirms the model's stability and minimal overfitting.

**
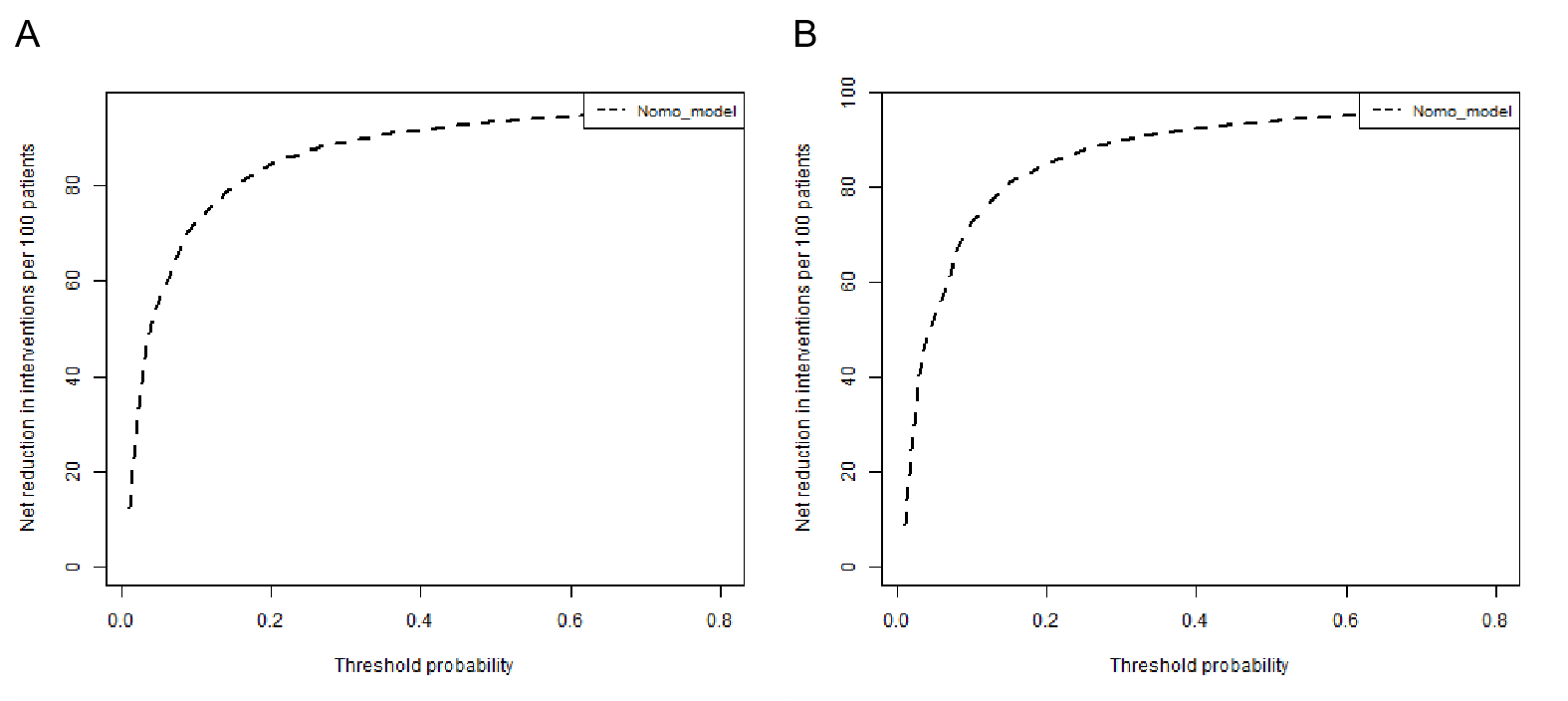
**

**Supplementary Figure 2** Intervention reduction analysis of the nomogram.

(A) Training cohort, (B) Validation cohort.
