# Supplementary material for: Development and validation of a nomogram-based prediction model for hospital-acquired carbapenem-resistant Acinetobacter baumannii in critically ill patients: a multicenter retrospective cohort study
Source: Front Cell Infect Microbiol. 2025 Nov 26;15:1679272. doi: 10.3389/fcimb.2025.1679272 (PMC12689862; doi:10.3389/fcimb.2025.1679272)
Supplement: Supplementary file 2 [file Table2.docx]

# =============================================================================

# ENHANCED REPLICATION CODE FOR CRAB PREDICTION MODEL

# =============================================================================

# 1. FINAL MODEL COEFFICIENTS (Verified against manuscript Table X)

model_coefficients <- c(

"(Intercept)" = -4.312,

"Carbapenem_exposure" = 0.891,

"Other_MDROs" = 0.764,

"Mechanical_ventilation" = 0.693,

"ICU_admissions" = 0.521,

"ICU_length_of_stay" = 0.004,

"Hospital_length_of_stay" = 0.002

)

# 2. ENHANCED PREDICTION FUNCTION

#' @title Predict Hospital-Acquired CRAB Risk

#' @description Complete implementation of the final logistic regression model

#' @param new_data Data.frame with required predictors (see details)

#' @return Data.frame with predictions and risk classification

#' @examples

#' # See Section 4 for example usage

predict_CRAB_risk <- function(new_data) {

tryCatch({

# Input validation

required_vars <- c("Carbapenem_exposure", "Other_MDROs", "Mechanical_ventilation",

"ICU_admissions", "ICU_length_of_stay", "Hospital_length_of_stay")

if (!all(required_vars %in% names(new_data))) {

stop("Missing required variables in input data")

}

# Enhanced value checks

validate_binary <- function(x, name) {

if (!all(x %in% c(0,1))) stop(paste(name, "must be 0 or 1"))

}

validate_binary(new_data$Carbapenem_exposure, "Carbapenem_exposure")

validate_binary(new_data$Other_MDROs, "Other_MDROs")

validate_binary(new_data$Mechanical_ventilation, "Mechanical_ventilation")

if (any(new_data$ICU_admissions < 1))

stop("ICU_admissions must be ≥1")

if (any(new_data$ICU_length_of_stay < 0))

stop("ICU_length_of_stay cannot be negative")

if (any(new_data$Hospital_length_of_stay < 0))

stop("Hospital_length_of_stay cannot be negative")

# Calculate linear predictor

linear_predictor <- model_coefficients["(Intercept)"] +

sum(model_coefficients[-1] * sapply(names(model_coefficients)[-1],

function(x) new_data[[x]]))

# Convert to probability

predicted_prob <- exp(linear_predictor) / (1 + exp(linear_predictor))

# Risk classification

risk_class <- ifelse(predicted_prob > 0.022, "High Risk", "Low Risk")

# Prepare output

result_df <- data.frame(

Predicted_Probability = round(predicted_prob, 4),

Risk_Classification = risk_class,

stringsAsFactors = FALSE

)

if ("Patient_ID" %in% names(new_data)) {

result_df <- cbind(Patient_ID = new_data$Patient_ID, result_df)

}

return(result_df)

}, error = function(e) {

message("Prediction Error: ", e$message)

return(data.frame(Error = e$message))

})

}

# 3. COMPREHENSIVE EXAMPLE USAGE

test_patients <- data.frame(

Patient_ID = c("Example_01", "Example_02", "Boundary_01"),

Carbapenem_exposure = c(1, 0, 1),

Other_MDROs = c(0, 0, 1),

Mechanical_ventilation = c(1, 0, 0),

ICU_admissions = c(2, 1, 5),

ICU_length_of_stay = c(10, 5, 0),

Hospital_length_of_stay = c(20, 10, 365)

)

predictions <- predict_CRAB_risk(test_patients)

print("Test Predictions:")

print(predictions)

# 4. EXPORT FUNCTIONALITY

write.csv(predictions, "CRAB_risk_predictions.csv", row.names = FALSE)
